# Supplementary material for: Taenia solium TAF6 and TAF9 bind to a downstream promoter element present in the Tstbp1 gene core promoter
Source: PLoS One. 2024 Aug 29;19(8):e0306633. doi: 10.1371/journal.pone.0306633 (PMC11361659; doi:10.1371/journal.pone.0306633)
Supplement: S1 Fig — Numbers to the right corresponds to nucleotide or amino acids respectively (GenBank: PP763292). (PDF) [file pone.0306633.s001.pdf]

```
cDNA  ATGGACGGGTTCGAGCAACGTCCTTGTGATCAGTTGTCGGTTTTAAGCGTAATCAAGTCA   60
Prot   M  D  G  F  E  Q  R  P  C  D  Q  L  S  V  L  S  V  I  K  S    20

cDNA  ATTTTTGACGATTTTAATTTGGCCGATTTATCTGAGGATGTCTATAATCATGTGATGGAT  120
Prot   I  F  D  D  F  N  L  A  D  L  S  E  D  V  Y  N  H  V  M  D    40

cDNA  ATTATTTCAAAATACACTGGTGAAATACTCGTCGATGCTAAATATAATGCTCTGTATGCC  180
Prot   I  I  S  K  Y  T  G  E  I  L  V  D  A  K  Y  N  A  L  Y  A    60

cDNA  GGGCGGTCAAATATTTCTGAACAAGATCTAGATTTGGCCGTTGAAAACAAGCTGGAAAAC  240
Prot   G  R  S  N  I  S  E  Q  D  L  D  L  A  V  E  N  K  L  E  N    80

cDNA  GTTATACTTGCGCCACTTCACAGGGGGCAACTTCTCGAATATGCCGAGAAAATCAATTCC  300
Prot   V  I  L  A  P  L  H  R  G  Q  L  L  E  Y  A  E  K  I  N  S   100

cDNA  CATGCTCTTCCGTCAATTAAGTCTGGACCAGGAATAAAGTTAGCTCCGGAGAAGTATACG  360
Prot   H  A  L  P  S  I  K  S  G  P  G  I  K  L  A  P  E  K  Y  T   120

cDNA  ATCACGGCGCCGAACTATTGTATTGCCTCAAATACTAGCTCAAATGCAACTTTCGTAAAT  420
Prot   I  T  A  P  N  Y  C  I  A  S  N  T  S  S  N  A  T  F  V  N   140

cDNA  GTGTCTGGCTCTATGAATATGTCCTCCAGAATCGTACTTCCCAGTTCAAATACAAGCGCT  480
Prot   V  S  G  S  M  N  M  S  S  R  I  V  L  P  S  S  N  T  S  A   160

cDNA  AGTTCTGGTAGTCTAGCGGTTTACCGTGTTTCAAATACACCTGGCAGTAATCCACAAGGT  540
Prot   S  S  G  S  L  A  V  Y  R  V  S  N  T  P  G  S  N  P  Q  G   180

cDNA  CAGCGTGGAACCGTGATGTCCTCATCTTCTGCTAATTCGGGTACGCTATCAGACTTGCCG  600
Prot   Q  R  G  T  V  M  S  S  S  S  A  N  S  G  T  L  S  D  L  P   200

cDNA  GGCATCAAATCTGCGCCGGCAGTTTTCGGAAGAGTAGATGTGCCATCCATCGCTGTAGTG  660
Prot   G  I  K  S  A  P  A  V  F  G  R  V  D  V  P  S  I  A  V  V   220

cDNA  GATACTTTATATCGTATAAATATCATTCAGATAATCATTCTTGTTCCTTACCTTATTGAA  720
Prot   D  T  L  Y  R  I  N  I  I  Q  I  I  I  L  V  P  Y  L  I  E   240

cDNA  GGATTTTAG                                                     729
Prot   G  F  -                                                      242
```

Supplementary Figure 1. TsTAF9 cDNA and protein sequence obtained by PCR amplification from a cDNA *T. solium* cyst library. Numbers to the right corresponds to nucleotide or amino acids respectively (GenBank: PP763292).
