# Supplementary material for: Taenia solium TAF6 and TAF9 bind to a downstream promoter element present in the Tstbp1 gene core promoter
Source: PLoS One. 2024 Aug 29;19(8):e0306633. doi: 10.1371/journal.pone.0306633 (PMC11361659; doi:10.1371/journal.pone.0306633)
Supplement: S2 Fig — Numbers to the right corresponds to nucleotide or amino acids respectively (GenBank: KY124274.1). (PDF) [file pone.0306633.s002.pdf]

cDNA ATGTTTTCGGAAGAGCGAAAGAAGCTAAACCGGCTCTCCCGCAAGGTCTCCAACCCCTGT 60
Prot M F S E E R K K L N R L S R K V S N P C 20

cDNA GGGGCCAAAAAACGCCTTCTTAACAGCCGCCTGGCCGGAAAATCCTCCGTAAAGGGCCAG 120
Prot G A K K R L L N S R L A G K S S V K G Q 40

cDNA AAACACCCTAGTAATAATGGCATCCATTCCTCCCGTTCCACAACTTCGAGACATCTCGAT 180
Prot K H P S N N G I H S S R S T T S R H L D 60

cDNA TTTCCCCTCGAGTTTTTTAAATCCTGTGCTGAAATTAATGCTGTCAGTCCCTTCTCGGGA 240
Prot F P L E F F K S C A E I N A V S P F S G 80

cDNA GAGGGTATTGGCGTGCTACAGAGGCACCTTCATCAAATCACTACCGCCCTTGTACAGAAC 300
Prot E G I G V L Q R H L H Q I T T A L V Q N 100

cDNA GCCGTTCGTAACATGGAGCAGAATCGGCGTGGTACTCCCCACATCTCCGACATCGATTCT 360
Prot A V R N M E Q N R R G T P H I S D I D S 120

cDNA GCGGCTCTGGCGATGGGCATGGACATTCCCTATGGTGCAGCTACTGGTGAACTTATCCCG 420
Prot A A L A M G M D I P Y G A A T G E L I P 140

cDNA GTAAGGACTAGCGGCCGCAACGCTGCTCCTGGAGTGGGTGGAAAGATGATCTTGATTCGA 480
Prot V R T S G R N A A P G V G G K M I L I R 160

cDNA AAAGATAAGGAGGTGGATATTAAGACCCTTCTTCGACGCCAACCCACGCCTGTTGTCTAC 540
Prot K D K E V D I K T L L R R Q P T P V V Y 180

cDNA GACATTAGTCTAGTGGTCCACTGGTTGGCGATAGATGGAGTTCAGCCAACCTCACCGCAG 600
Prot D I S L V V H W L A I D G V Q P T S P Q 200

cDNA AATCCACCACCAGAGTTTCTGCGTCGAATGATTATACTTTCAGGCACTCAGACGCCAAAG 660
Prot N P P P E F L R R M I I L S G T Q T P K 220

cDNA GCAATCTGTACAGCTCTAAACCCCACTATAAAAGTCGACGACACGCAGCATCAGCCCGTT 720
Prot A I C T A L N P T I K V D D T Q H Q P V 240

cDNA GACGCAAAGATGGATAAGAACAAAGTGGGGGACGATGGAGTTTCTCATCCGCGGGTTATG 780
Prot D A K M D K N K V G D D G V S H P R V M 260

cDNA CAAGCCCTTCATGTTGAACGGCGTCCGCAAGAAGTCAGTCAAGAGTTGATGCTCTATTTT 840
Prot Q A L H V E R R P Q E V S Q E L M L Y F 280

cDNA CGAGAGCTCACTGAAGCCTGCGTCGGAGCCAATGAGATCCGTCGGCGAGACGCGCTGGAA 900
Prot R E L T E A C V G A N E I R R R D A L E 300

cDNA AATGCCACCCTAGACACGGGTCTGCAACCCCTTGTGCCTTACCTCGTCACCTTCATTGCT 960
Prot N A T L D T G L Q P L V P Y L V T F I A 320

cDNA GAGGGTATCCGGTTGAATGCGATAAACAGCAATCTGGCTATCCTTATCTACCTGGTGCGT 1020
Prot E G I R L N A I N S N L A I L I Y L V R 340

```
cDNA CTGACCAAGGCTTTGGTTGACAATCCCAACGTCACCCTGAAGGCCTACTTGCAGAGCCTT 1080
Prot  L  T  K  A  L  V  D  N  P  N  V  T  L  K  A  Y  L  Q  S  L   360

cDNA GTGCCAGGCATCATCACTTGTAGCCTCTGTCGCCAGGTCTGCGCTAAACCCATCACCGAC 1140
Prot  V  P  G  I  I  T  C  S  L  C  R  Q  V  C  A  K  P  I  T  D   380

cDNA AACCACTGGGCGCTACGTGATTTTGCGGCCAAGCAACTCGTCGCCATCTGTAACAAATAC 1200
Prot  N  H  W  A  L  R  D  F  A  A  K  Q  L  V  A  I  C  N  K  Y   400

cDNA AACACCTCGTGCAACGGTCTCTACAGTCGCATAACACGCGAACTCTACCGCGTGCTGTCC 1260
Prot  N  T  S  C  N  G  L  Y  S  R  I  T  R  E  L  Y  R  V  L  S   420

cDNA GCTTGGATTGAGGGCAAATCGGCAGCCACTTCGGACCACTTTTGCGCCTCCGCTTCCTCT 1320
Prot  A  W  I  E  G  K  S  A  A  T  S  D  H  F  C  A  S  A  S  S   440

cDNA TCTGTTGCCACCACATCTGCCACTACCGACAACACTGACCACTCTTCCACCGCTGCACCC 1380
Prot  S  V  A  T  T  S  A  T  T  D  N  T  D  H  S  S  T  A  A  P   460

cDNA ACCGATCCTGCACGCAAGGAGATCGCAGGGGTGCCCCGAGTTTCCCTGGGCATGGCAGTA 1440
Prot  T  D  P  A  R  K  E  I  A  G  V  P  R  V  S  L  G  M  A  V   480

cDNA GACTCTCTCAACACTCTCTACGGCACCCTCACCTGTATCACCGAGTTTGGTGGCAATTGC 1500
Prot  D  S  L  N  T  L  Y  G  T  L  T  C  I  T  E  F  G  G  N  C   500

cDNA CTCCGCATGCTTGTCTTTCCTCGCCTACCAGCGCTGTGCAGACGCCTCACTCGTATGACC 1560
Prot  L  R  M  L  V  F  P  R  L  P  A  L  C  R  R  L  T  R  M  T   520

cDNA ACTGCTTCTGCATCCGTTGCTTCACAATCCACTGCATCAGTGTCAAATTCAGCGGAGATG 1620
Prot  T  A  S  A  S  V  A  S  Q  S  T  A  S  V  S  N  S  A  E  M   540

cDNA GTGATGGTAATGGACCAGGAGGGCTTTCAGCAAACCCTCAACGCCTCTTCCACTACCACT 1680
Prot  V  M  V  M  D  Q  E  G  F  Q  Q  T  L  N  A  S  S  T  T  T   560

cDNA CTACTTTCCAACGCTGAAATGAGATCGTTGGACTCGCTTAAGAAACTTATGAACACGCGA 1740
Prot  L  L  S  N  A  E  M  R  S  L  D  S  L  K  K  L  M  N  T  R   580

cDNA TTCGTGAGTCTATTGGCGGAGTGGCGAGTAAGGCAGAATCTGCCAGTGACTCTGGAGGCC 1800
Prot  F  V  S  L  L  A  E  W  R  V  R  Q  N  L  P  V  T  L  E  A   600

cDNA TACAAAGCGGACTACGGAATTATGGCGTCGTGTCTGTTCGCGTTGGCGGCCCCGGCAGAC 1860
Prot  Y  K  A  D  Y  G  I  M  A  S  C  L  F  A  L  A  A  P  A  D   620

cDNA CATCCGCGTCCTCATGCGACCCCTGCGGGGGCGCTTATTGTGACTAAACCACAGCCTCCC 1920
Prot  H  P  R  P  H  A  T  P  A  G  A  L  I  V  T  K  P  Q  P  P   640

cDNA CTCAATGTCTCCCACTAG                                           1938
Prot  L  N  V  S  H  -                                             645
```

Supplementary Figure 2. TsTAF6 cDNA and protein sequence obtained by PCR amplification from a cDNA *T. solium* cyst library. Numbers to the right corresponds to nucleotide or amino acids respectively (GenBank: KY124274.1).
