# Supplementary material for: Taenia solium TAF6 and TAF9 bind to a downstream promoter element present in the Tstbp1 gene core promoter
Source: PLoS One. 2024 Aug 29;19(8):e0306633. doi: 10.1371/journal.pone.0306633 (PMC11361659; doi:10.1371/journal.pone.0306633)
Supplement: S3 Fig — Alignment comparison for amino acid sequence A) TAF9 from Homo sapiens (HsTAF9) and T. solium (TsTAF9, from GenBank: PP763292). B) TAF6 from Homo sapiens (HsTAF6) and T. solium (TsTAF6, GenBank: KY124274.1). In yellow is highlighted the region used as immunogen for antibodies development according to manufacturer. Underlined amino acids represents the epitopes for both species, predicted using BCPREDS web server (http://ailab-projects2.ist.psu.edu/bcpred/predict.html). Asterisks (*) denotes identic amino acids and colon (:) denotes homologous amino acids. (PDF) [file pone.0306633.s003.pdf]

A

```
HsTAF9    MESGKTASPKSMPKDAQMMAQILKDMGITEYEPRVINQMLEFAFRYVTTILDDAKIYSSH   60
TsTAF9    -MDGFEQRPCDQLSVLSVIKSIFDDFNLADLSEDVYNHVMDIISKYTGEILVDAKYNALY   59
             *    *        ::  *: *: :::    * *:::::  :*   ** ***  : :

HsTAF9    AKKATVDADDVRLAIQCRADQSFTSPPPRDFLLDIARQRNQTPLPLIKPYSGPRLPPDRY  120
TsTAF9    AGRSNISEQDLDLAVENKLENVILAPLHRGQLLEYAEKINSHALPSIKSGPGIKLAPEKY  119
          * :: :  :*: **:: : :: : :*  *  **: * : *   ** **   * :* *::*

HsTAF9    CLTAPNYRLKSLQKKASTSA---------GRITVPRLSV----GS-----VTSRPSTPTL  162
TsTAF9    TITAPNYCIASNTSSNATFVNVSGSMNMSSRIVLPSSNTSASSGSLAVYRVSNTPGSNPQ  179
           :***** : *     :*            ** :*        **     *:  * :

HsTAF9    GTPTPQTMSVSTKVGTPMSLTGQRFTVQM-PTSQSP--AVKASIPATSAVQNVLINPSLI  219
TsTAF9    GQRGTVMSSSSANSGTLSDLPGIKSAPAVFGRVDVPSIAVVDTLYRINIIQIIILVPYLI  239
          *       * *:: **   * * : :  :    : *  **  ::     :* ::: * **

HsTAF9    GSKNIFITTNMMSSQNTANESSNALKRKREDDDDDDDDDDDDYDNL                264
TsTAF9    EGF-------------------------------------------                242
              : : :    * * :**   *:*:  ::
```

B

```
HsTAF6    ALKLKNVEPLYGFHAQEFIPFRFA------SGGGRELYFYEEKEVDLSDIINTPLPRVPL  127
TsTAF6    AALAMGMDIPYGAATGELIPVRTSGRNAAPGVGGKMILIRKDKEVDIKTLLRRQPTPVVY  180
          *     ::  **  : *:** * :        **: : : ::****:  ::      *

HsTAF6    DVCLKAHWLSIEGCQPAIPENPPPAPKEQQKAEATE--P------L-KSAKPGQEEDGPL  178
TsTAF6    DISLVVHWLAIDGVQPTSPQNPPPEFLRRMIILSGTQTPKAICTALNPTIKVDDTQHQPV  240
          *: *  ***:*:* **: *:****    :    :    *      *  : *  : :  *:

HsTAF6    KGKGQGATTADGKGKE-KKAPPLLEGAPLRLKPRSIHELSVEQQLYYKEITEACVGSCEA  237
TsTAF6    DAKMDKN----KVGDDGVSHPRVMQALHV---ERRPQEVSQELMLYFRELTEACVGANEI  293
            * :        * :    * :::   :    *  :*:* *  **::*:******: *

HsTAF6    KRAEALQSIATDPGLYQMLPRFSTFISEGVRVNVVQNNLALLIYLMRMVKALMDNPTLYL  297
TsTAF6    RRRDALENATLDTGLQPLVPYLVTFIAEGIRLNAINSNLAILIYLVRLTKALVDNPNVTL  353
          :* :**:  : * **  ::* : ***:**:*:* :: ***:****:*: ***:*** : *
```

Supplementary Figure 3. Alignment comparison for amino acid sequence A) TAF9 from Homo sapiens (HsTAF9) and *T. solium* (TsTAF9, from GenBank: PP763292). B) TAF6 from *Homo sapiens* (HsTAF6) and *T. solium* (TsTAF6, GenBank: KY124274.1). In yellow is highlighted the region used as immunogen for antibodies development according to manufacturer. Underlined amino acids represents the epitopes for both species, predicted using BCPREDS web server (http://ailab-projects2.ist.psu.edu/bcpred/predict.html). Asterisks (*) denotes identic amino acids and colon (:) denotes homologous amino acids.
