# Supplementary figures and images for: Taenia solium TAF6 and TAF9 bind to a downstream promoter element present in the Tstbp1 gene core promoter
Source: PLoS One. 2024 Aug 29;19(8):e0306633. doi: 10.1371/journal.pone.0306633 (PMC11361659; doi:10.1371/journal.pone.0306633)

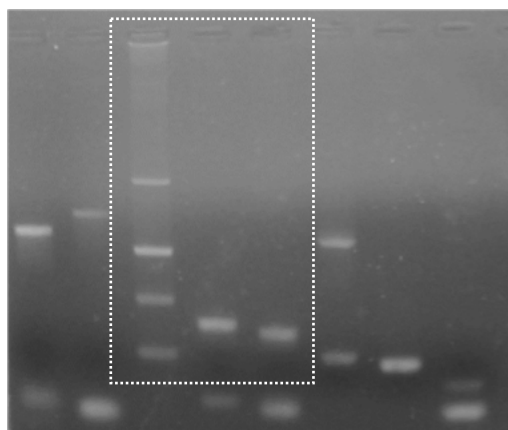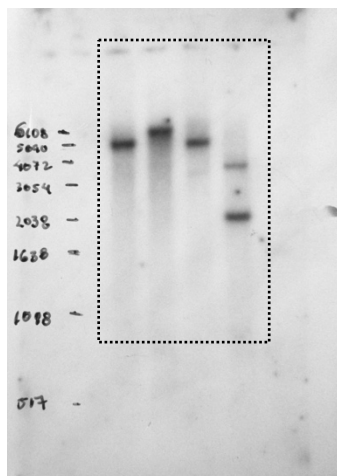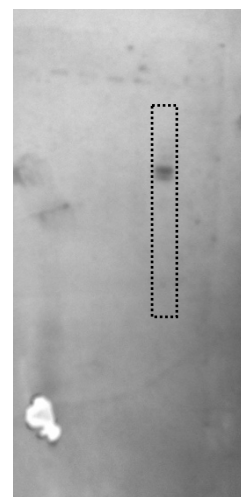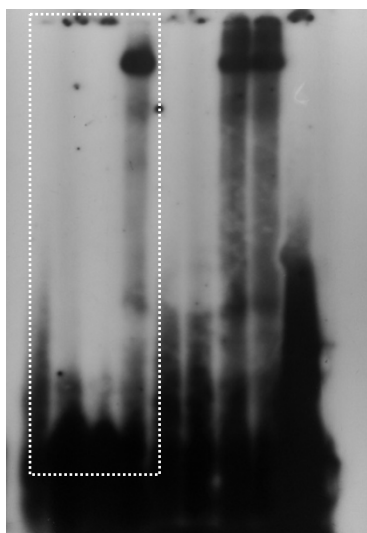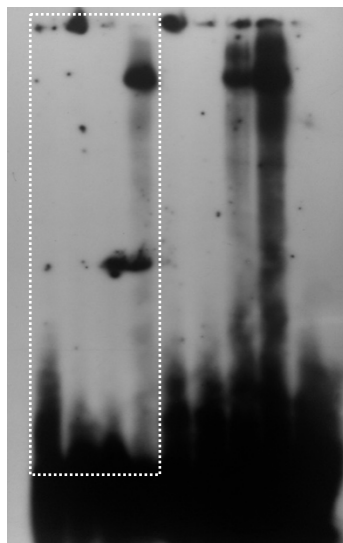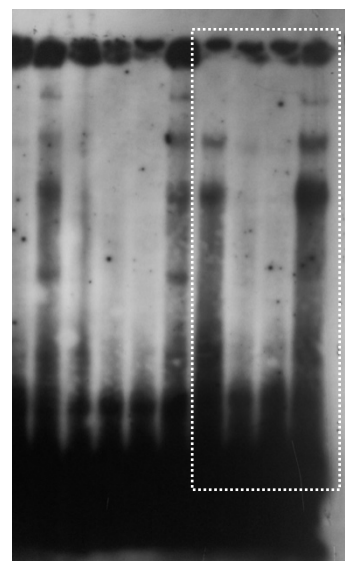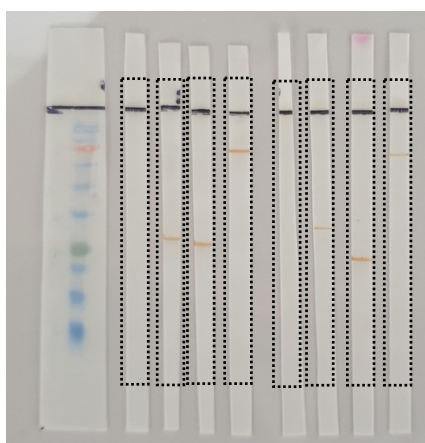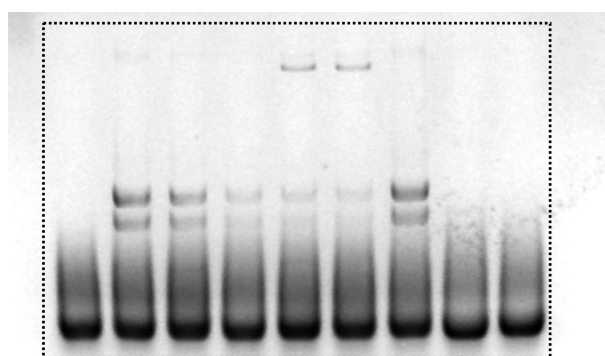

**S1\_raw\_images.** Uncropped blots from Figures 3, 5, 6 and 7. Boxes indicate where the blots were cropped.

Supplement: S1 Raw images — (PDF) [file pone.0306633.s004.pdf]
